# Design and fabrication of a 3D-printed drone-integrated winching system

**DOI:** 10.1016/j.ohx.2025.e00642

**Published:** 2025-03-18

**Authors:** William Crowe, Benicio Costales, Kyle Luthy

**Affiliations:** Wake Forest University, 1834 Wake Forest Rd, Winston-Salem, NC 27109, United States

**Keywords:** Winch, Drone, Remote, Aerial, Environmental Sampling

## Abstract

Existing multi-rotor drone-based winching platforms can be expensive, complex, proprietary, and heavy. Here, an inexpensive, simple, effective, and lightweight drone winching platform named the CLARK-3 is described. This winching platform is suitable for payloads of up to 3 kg in its existing configuration and relies heavily on readily available standard parts and 3D printed components while being compatible with a variety of drone controllers. The CLARK-3 in its existing configuration weighs 0.8 kg, can hold up to 27 m of 130 N monofilament, and has a low power consumption at maximum load (∼16 W). Considerations were made during the design of the CLARK-3 to ensure field serviceability by reducing part count and simplifying disassembly.

## Highlights

• 3 kg load rated drone winch built from 3D-printed parts and off-the-shelf materials.
• Drone winch integration with MavLink provides line length to operator.
• Drone winch controlled via standard pulse width modulated signal.
• Drone winch with integrated limit switch.

## Graphical abstract

**Figure:**
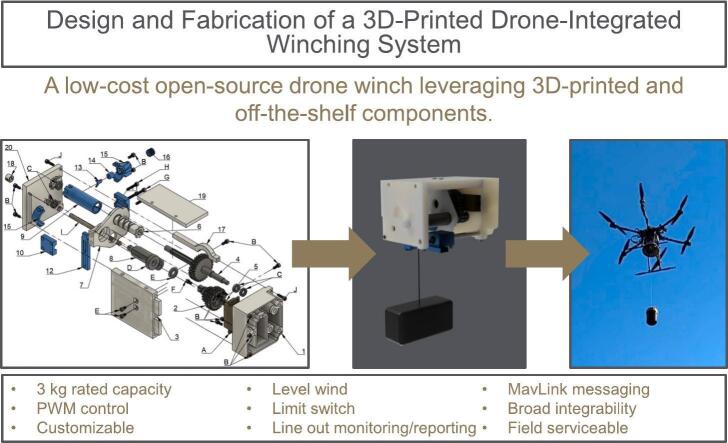

Specifications table.

**Table:** 

Hardware name	*Customizable Light-duty Aerial Recovery Kit (CLARK-3)*
Subject area	General
Hardware type	Field measurements and sensors
Closest commercial analog	*The Daiwa winch from Okaya & Co., LTD.*
Open source license	*Hardware (mechanical and electrical designs) are licensed under the CERN-OHL-S V2.0 and software is licensed under LGPL*
Cost of hardware	*Approximately $120.00 (USD)*
Source file repository	https://doi.org/10.17632/pd67rx3k5y.1

## 1 Hardware in context

The ubiquity of drones has made them an ideal platform for environmental sensing and sampling applications where they can provide access to remote, hazardous, or hard-to-reach locations. Some systems use the drone for direct environmental contact and sampling, such as for algae [1] and ice sampling from icebergs [2]. Other activities require the payload to be located away from the drone itself either to avoid effects of propwash or to provide access at some distance below the drone where it cannot safely maneuver (e.g., within a forest canopy [3]). In some instances, the payload is suspended below the drone at a fixed distance. This is seen in many water sampling studies using payloads such as Niskin bottles and water collection sleeves [4], [5], [6], [7]. As the desired sampling depth increases, so will the length of the suspension line. Others have leveraged winching systems to provide a means to position the payload when on site rather than flying with a lengthy suspended pendulum. The LUSI drone [8] used a custom winch and payload to obtain mud samples from Lusi mud eruption in Indonesia, Ryu [9] developed a winch and open-source payload for water sampling and sensing, Poulsen et al. [10] collect oceanographic profiles, and Montoya et al. [11] developed a winch to test gas concentrations over landfills. The effort described here provides an accessible and customizable 3D-printable baseline winch design to support research efforts, environmental or otherwise. This open-source effort will be referred to as the CLARK-3 (Customizable Light-duty Aerial Recovery Kit – 3 kg).

Additive manufacturing techniques are gaining popularity for the production of custom drones and drone systems. Several research groups have developed 3D printed drone airframes, some leveraging common materials used in Fused Deposition Modeling (FDM) such as polylactic acid (PLA) [12], [13], while others adopt more exotic composites, such as carbon and glass fiber reinforced materials, to increase overall strength and reduce weight while allowing for more complex geometries [14], [15], [16]. The system described herein relies primarily upon PLA due to the accessibility of this material, as PLA is widely supported across 3D printer manufacturers. There still exist a number of challenges for additive manufacturing of drones (i.e. printer build volume) [17] and material choices and manufacturing methods are also important considerations in the developing landscape of sustainability throughout the drone lifecycle [18].

Drone-based winching systems are increasingly popular for delivery services such as those provided by Walmart [19], Wing [20] and ZipLine [21]. These are typically proprietary and integrated into use-specific drones. There are a few winching systems that are commercially available such as Okaya’s Daiwa winch [22], FoxTech’s F10 winch [23], ViewPro winch [24] and A2Z Drone Delivery System’s Rapid Delivery System 2 (RDS2) [25]. These winch systems weigh between 630–1500 g and can lift a payload up to 5 kg. The working length is 30 m for all but the Daiwa winch which supports 80 m. Most employ a level wind system to prevent line fouling. The control scheme varies, and some are independent (separate radio) while others can be integrated into existing interfaces such as those available through DJI [26] or MAVLink [27]. Not all prices are published, but those that are are on the order of $2,500 USD. These systems were used to establish the following design requirements for the CLARK-3 (see Table 1 for comparison):

• The system must be small and have a customizable mounting system to support various airframes.
• The system must be able to mitigate line fouling (level-wind).
• The system should be able to be integrated directly with a platform’s power and communication system.
• The system should be able to provide feedback regarding the length of line deployed.
• The system should be able to lift at least a 3 kg payload (equivalent to 3L of fresh water).

**Table 1:** Comparison of characteristics of commercially available winching systems with the CLARK-3 winch system.

**Winch**	Daiwa [22]	F10 [23]	ViewPro [24]	RDS2 [25]	CLARK-3
**Weight**	0.63 kg	1.1 kg	1.05 kg	1.5 kg	0.8 kg*
**Dimensions**	110x82x72 mm	177x110x103 mm	200x105x145 mm	205x145x105 mm	141x95x85 mm^#^
**Payload Lift Weight**	5 kg	5 kg	5 kg	5 kg	3 kg
**Line Length**	80 *m*	30 *m*	30 *m*	30.48 *m*	27 *m*
**Control**	Independent or integrated (DJI, ArduPilot)	Independent remote	Independent remote	Integrated (MavLink)	Integrated (MavLink)
**Level Wind**	Yes	Yes	Yes	Unknown	Yes

*weight of the CLARK-3 depends on 3D printed infill percentage, sample container, and accessory use. Range is 0.6–1.1 kg in typical conditions.

^#^dimensions given of primary winching platform without sampling container collector or drone mount attached.

While commercial alternatives (Table 2) exist, the open-source CLARK-3 winching platform provides some unique advantages. By being open-source and using readily available components and common additive manufacturing techniques, the CLARK-3 is customizable to user specific needs. Mounting options, gear ratios, and control software (i.e. allowable speeds, drone interfacing, and line limits) are all tunable. Being open also allows for rapid, low-cost repair of the winch and the production of spare parts, which makes it field serviceable. The choice of materials also results in a considerable cost reduction and manufacturing accessibility, with the CLARK-3 costing roughly $120 USD. Keeping the cost low makes this an attractive system to use for hazardous tasks where there is the possibility that the system will be lost or damaged. Ultimately, by being low-cost and customizable, the CLARK-3 is intended to make aerial drone based winching tasks more accessible for a wider range of users. These objectives were met in the current design with future efforts focusing on increasing payload capacity and winching speed by further exploring motor options and gearing ratios.

**Table 2:** Description and repository links for all design files and supplemental assembly instructions.

**Design file name**	**File type**	**Open source license**	**Location of the file**
Winch_Electronics_Tray_Blank.step	CAD File	CERN-OPL-S	Mendeley Repository
Winch_LinearBearing_Block_Bottom.step	CAD File	CERN-OPL-S	Mendeley Repository
Winch_LinearBearing_Block_Top.step	CAD File	CERN-OPL-S	Mendeley Repository
Winch_Cable_Guide.step	CAD File	CERN-OPL-S	Mendeley Repository
Winch_Traveler_Bit.step	CAD File	CERN-OPL-S	Mendeley Repository
Winch_Bit_Holder.step	CAD File	CERN-OPL-S	Mendeley Repository
Winch_Traveler_Bit_Tensioner.step	CAD File	CERN-OPL-S	Mendeley Repository
Winch_Traveler_Guide_Bottom.step	CAD File	CERN-OPL-S	Mendeley Repository
Winch_Traveler_Guide_Top.step	CAD File	CERN-OPL-S	Mendeley Repository
Winch_Spool_Tip.step	CAD File	CERN-OPL-S	Mendeley Repository
Winch_Spool.step	CAD File	CERN-OPL-S	Mendeley Repository
Winch_Spool_Gear.step	CAD File	CERN-OPL-S	Mendeley Repository
Winch_Servo_Gear.step	CAD File	CERN-OPL-S	Mendeley Repository
Winch_Levelwind_Guide.step	CAD File	CERN-OPL-S	Mendeley Repository
Winch_Diamond_Gear.step	CAD File	CERN-OPL-S	Mendeley Repository
Winch_Levelwind_Gear.step	CAD File	CERN-OPL-S	Mendeley Repository
Winch_Backingplate.step	CAD File	CERN-OPL-S	Mendeley Repository
Winch_Geartrain_Support.step	CAD File	CERN-OPL-S	Mendeley Repository
Winch_Curved_Support.step	CAD File	CERN-OPL-S	Mendeley Repository
Winch_Center_Support.step	CAD File	CERN-OPL-S	Mendeley Repository
Winch_Servo_Housing.step	CAD File	CERN-OPL-S	Mendeley Repository
Winch_v4_Assembly v29.step	CAD File	CERN-OPL-S	Mendeley Repository
Winch_Assembly_Video_1.mp4	MP4 File	CERN-OPL-S	Mendeley Repository
CLARK3_Winch_Code_V1.0.ino	Arduino.ino file	LGPL	Mendeley Repository
Winch_electronics_schematic	.sch	CERN-OPL-S	Mendeley Repository
Winch_electronics_board	.brd	CERN-OPL-S	Mendeley Repository
Winch_electronics_Gerber_package	.zip	CERN-OPL-S	Mendeley Repository

## 2 Hardware description

The CLARK-3 open-source drone-based winching system is completely customizable to the user’s needs, using a continuous servo as the core of the platform rated at 25 kg*cm to reliably lift payloads of up to 3 kg as designed. This limitation is not a limit of the materials or hardware, but of the torque of the motor given the gear ratio provided. By manipulating the gear ratio, this winching system could support payloads over 10 kg. Many existing winching platforms cost more than $2500 USD, however the proposed CLARK-3 platform would cost an average user between $75 and $120 USD, depending on materials used. This low cost is enabled by using common off-the-shelf hardware, bearings, and a continuous servo motor as well as low-cost 3D printed parts. Because of this approach, no machining or otherwise labor-intensive components are required. While many of the existing solutions are pre-built and require little set up, the CLARK-3 only requires minimal tools that many research labs may already have, about 12–20 h of print time depending on the printer and configuration, and 1–2 h to build and set up. Anyone familiar with 3D printing and post-processing will already have the tools necessary for preparing the parts and assembly requires only common tools and can be completed by an undergraduate student in little time. Should higher strength or durability be required, changes in material or design can be implemented to improve the characteristics of the winch as needed. The design of the CLARK-3 emphasizes user serviceability. Once fabricated, part replacement can be carried out in the field quickly and without special tools. The gearing of the CLARK-3 uses a herringbone tooth pattern to reduce thrust force while maintaining excellent strength and quiet operation.

Most of the CLARK-3 structure is 3D printed in Polylactic Acid (PLA) using Fused Deposition Modeling (FDM). To aid in mitigating line fouling, the system uses a diamond gear, which allows a guide to lay the line back and forth along the drum. To achieve higher accuracy and a smoother surface, this diamond gear is printed using a stereolithography (SLA) printer. In addition to the continuous servo motor, the system relies on readily available components including a metal guide rod, some bearings, screws, and screw inserts.

The CLARK-3 is controlled by an Arduino Pro Mini due to the Arduino’s ease of use, customization potential, and readily available software. The Arduino Pro Mini controls the winching system based on pulse width modulated (PWM) input from the drone flight controller (in this case, a Pixhawk 2). Feedback is provided from a limit switch to detect when the payload is in contact with the winching platform. A Hall effect sensor monitors the winch spool motion and provides a deployed line estimate. Any variability in the measured signals can be accounted for and refined for a user’s specific sensors and flight controllers by updating the Arduino software. While the version of this platform described here relied on a Pixhawk 2 flight controller, most flight controllers which can deliver PWM output to a custom device may be used for control of the winching system. The hall effect sensor used for this work was a digital unipolar sensor, to simplify design and code.

The advantages of the CLARK-3 over similar winching platforms on the market are:

• Customizable design for variable payload weight, line speed, and sensing packages
• Controllable via simple PWM control, can be integrated with most drone platforms
• Small overall size with low weight
• Only 3D printed parts, off the shelf components, and simple tools required for fabrication
• Easily serviceable for user maintenance or modification
• Maximum payload as delivered: 3 kg (Up to 10 kg possible with gear ratio adjustment)

## 3 Design files summary

Winch_Electronics_Tray_Blank.step.

This file attaches a removeable tray to the rear of the winch assembly. This tray comes blank but can be modified to support any electronics you may want to attach to the winch assembly. As assembled, heat inserts were used to mount the electronics as well as a cover.

Winch_LinearBearing_Block_Top.step.

Winch_LinearBearing_Block_Bottom.step.

These two files are the top and bottom of a bearing block that allows tattachment of the linear bearing to the cable guide of the traveler assembly on the levelwind.

Winch_Cable_Guide.step.

This component attaches between the linear bearing block and the traveler bit guide and serves to guide the cable across the spool.

Winch_Traveler_Bit.step.

This component is the actual part that interacts with the diamond screw to move the cable guide back and forth along the spool. This part typically requires replacement every 0.5–1 km of cable travel.

Winch_Bit_Holder.step.

This component constrains the bit within the traveler bit guide while allowing it to rotate to follow the diamond screw threads.

Winch_Traveler_Bit_Tensioner.step.

This component compresses a spring behind the traveler bit holder, increasing the force engaging the bit with the diamond screw.

Winch_Traveler_BitGuide_Top.step.

Winch_Traveler_BitGuide_Bottom.step.

These components make up the guide that constrains the motion of the diamond screw as the traveler bit rides in the diamond screw.

Winch_Spool_Tip.step.

This component covers the tip of the spool as it exits the frame backplate and houses a magnet that interacts with the hall effect sensor to measure winch payout.

Winch_Spool.step.

This component is driven by the spool gear and is the component the cable or line winds around to raise or lower the payload.

Winch_Spool_Gear.step.

This component is driven by the servo gear and drives the spool to raise or lower the payload.

Winch_Servo_Gear.step.

This component is driven by the servo and serves to distribute power to both the levelwind gear and the spool gear.

Winch_Levelwind_Guide.step.

This component constrains the levelwind bit guide to move in a linear fashion and reduces the amount of debris that can interact with the levelwind bit.

Winch_Diamond_Gear.step.

This component allows the levelwind mechanism to function as a bi-directional screw mechanism where the levelwind moves in both linear directions given a single rotational direction.

Winch_Backingplate.step.

This component constrains all of the shafts of the mechanism together on the levelwind side of the frame and provides a location for the hall effect sensor to mount.

Winch_Geartrain_Support.step.

This component separates the gearbox from the levelwind and spool, it also houses the bearings supporting the geartrain and maintains alignment of the rotational components.

Winch_Curved_Support.step.

This component provided structural support across the bottom of the winching system. The curved section of this support is important to prevent interference with the levelwind gear.

Winch_Center_Support.step.

This component connects the servo housing to the backplate and provides structural support to the system and positions the geartrain support.

Winch_Servo_Housing.step.

This component holds the servo and bearings for the geartrain.

Winch_v4_Assembly v29.step.

This is the assembly file of all of the individual components previously listed.

Winch_Assembly_Video_1.mp4.

This is an instructional video that walks through the steps to assemble the mechanical components of the winch.

CLARK3_Winch_Code_V1.0.ino.

Arduino code to control winch direction, assess the limit switch, and handle the count of spool rotations for line payout monitoring.

Winch_Electronics_Schematic.sch.

Schematic of the winch control electronics created in Fusion 3D.

Winch_Electronics_Board.brd.

Board layout of the winch control electronics created in Fusion 3D.

Winch_Electronics_Gerber_Package.zip.

This zip file contains the Gerber files necessary to have the winch electronics board manufactured.

## 4 Bill of materials summary

### 4.1 Build instructions

#### 4.1.1 3D printing

The 3D printed components are listed in Table 3 along with their associated materials and print settings. All parts except for the diamond screw (6) were fabricated using an Ultimaker S3 loaded with PLA material (MAT-1). No Polyvinyl Alcohol (PVA) support material is necessary for fabrication to ease manufacturing by those without dual extruder printers, but some parts do require support. PLA material was selected for use here to improve the accessibility of this winching platform as well as for the high stiffness and relative ultimate strength that PLA offers. Other materials could be selected by the user to better suit their application, such as polycarbonate, but more flexible materials may not be suitable for this application. All fused deposition modeling (FDM) printed parts were completed with 1.2 mm wall thickness, 1.2 mm top/bottom thickness, and 20 % infill density in a tri-hexagonal pattern to balance part strength with print speed. Intricate parts (gears, traveler components) were printed at 0.1 mm layer height, all other FDM printed parts were printed at a 0.16 mm layer height. The diamond screw was printed using a Form 3 printer from FormLabs in white resin (MAT-2). Standard settings were used with a 0.05 mm layer height (Table 4).

**Table 3:** Bill of Materials for winch construction. *Note that the PLA Filament (MAT-1) and the White Resin (MAT-2) can support multiple builds but are only available in set quantities. Screws are also cheaper if purchased in kits rather than individual quantities and are therefore not listed with prices per unit. Therefore, the total cost of the BOM is higher than the actual cost of an individual unit (actual cost estimated to be ∼$120 USD).

**Designator**	**Component**	**Quantity**	**Cost per unit − [USD]**	**Total cost −** **[USD]**	**Source of materials**	**Material type**
A	Continuous Servo	1	$24.99	$24.99	Amazon	Other
B	M3 × 8 mm Hex Screws	10	See note in caption		Amazon	Metal
C	MR128-2RS Bearings (8 × 12 × 3.5 mm)	6	$0.99	$5.94	Amazon	Metal and Polymer
D	6900-2RS Bearings (10 × 22 × 6 mm)	1	$0.93	$0.93	Amazon	Metal
E	6700-2RS Bearings (10 × 15 × 4 mm)	1	$1.10	$1.10	Amazon	Metal and Polymer
F	M3 × 6 mm He× Screws	9	See note in caption			Metal
G	M3 × 14 mm He× Screws	4	See note in caption			Metal
H	M3 × 18 mm He× Screws	2	See note in caption			Metal
I	6 × 100 mm Linear Motion Shaft	1	$10.63	$10.63	McMaster-Carr	Metal
J	M3 × 12 mm He× Screws	2	See note in caption			Metal
K	M2.5 × 8 mm He× Screws	2	See note in caption			Metal
L	M2.5 Threaded Heat-Set Inserts for Plastic	1	14.11	14.11	McMaster-Carr	Metal
M	M3 Threaded Heat-Set Inserts for Plastic	1	21.02	21.02	McMaster-Carr	Metal
N	N52 1/8″ × 1/16″ Magnet	1	$0.32	$0.32	McMaster-Carr	Metal
O	LM6UU Linear Bearing	1	$0.93	$0.93	Amazon	Metal
P	0.6 × 7 × 10 mm Compression Spring	1	$1.19	$1.19	Amazon	Metal
MAT-1	PLA Filament	1	$20.87	$20.87	Matterhackers	Polymer
MAT-2	Formlabs White Resin V4	1	$149.00	$149.00	Formlabs	Polymer
PCB	Control and Interface PCB	1	26.57	26.57	PCBWay	Other
U1	7.5 V Step-Down Voltage Regulator	1	24.95	24.95	Pololu	Other
U2	Arduino Pro Mini 3.3 V	1	10.95	10.95	Sparkfun	Other
U3	Hall Effect Sensor − US5881LUA	1	2.00	2.00	Adafruit	Other
R1	10kOhm 1/4W Resistor	1	0.75	0.75	Adafruit	Other
SW1	Limit Switch KW12-3	1	5.99	5.99	Amazon	Other
J1	4 contact JST header B4B-XH-A	1	0.23	0.23	Digikey	Other
J1-A	4 contact JST receptacle XHP-4	1	0.11	0.11	Digikey	Other
J2	5 contact JST header B5B-XH-A	1	0.18	0.18	Digikey	Other
J2-A	5 contact JST receptacle XHP-5	1	0.13	0.13	Digikey	Other
J3	3 contact JST header B3B-XH-A	1	0.21	0.21	Digikey	Other
J3-A	3 contact JST receptacle XHP-3	1	0.11	0.11	Digikey	Other
J4	2 contact JST header B2B-XH-A	1	0.15	0.15	Digikey	Other
J4-A	2 contact JST receptacle XHP-2	1	0.10	0.10	Digikey	Other
			Total:	$351.26*

**Table 4:** Listing of 3D printed components.

**Designator**	**Component**	**Print Method/Material**	**Print Settings**
1	Servo Housing	FDM/PLA	Wall thickness: 1.2 mm Top/bottom thickness: 1.2 mm Infill density: 20 % Layer height: 0.16 mm
2	Servo Gear	FDM/PLA	Wall thickness: 1.2 mm Top/bottom thickness: 1.2 mm Infill density: 20 % Layer height: 0.1 mm
3	Center Frame Support	FDM/PLA	Wall thickness: 1.2 mm Top/bottom thickness: 1.2 mm Infill density: 20 % Layer height: 0.16 mm
4	Level Wind Gear	FDM/PLA	Wall thickness: 1.2 mm Top/bottom thickness: 1.2 mm Infill density: 20 % Layer height: 0.1 mm
5	Spool Gear	FDM/PLA	Wall thickness: 1.2 mm Top/bottom thickness: 1.2 mm Infill density: 20 % Layer height: 0.1 mm
6	Diamond Screw	SLA/Formlabs White Resin	Default
7	Geartrain Support	FDM/PLA	Wall thickness: 1.2 mm Top/bottom thickness: 1.2 mm Infill density: 20 % Layer height: 0.16 mm
8	Spool	FDM/PLA	Wall thickness: 1.2 mm Top/bottom thickness: 1.2 mm Infill density: 20 % Layer height: 0.16 mm
9	Level Wind Guide	FDM/PLA	Wall thickness: 1.2 mm Top/bottom thickness: 1.2 mm Infill density: 20 % Layer height: 0.16 mm
10	Linear Bearing Block 1	FDM/PLA	Wall thickness: 1.2 mm Top/bottom thickness: 1.2 mm Infill density: 20 % Layer height: 0.16 mm
11	Linear Bearing Block 2	FDM/PLA	Wall thickness: 1.2 mm Top/bottom thickness: 1.2 mm Infill density: 20 % Layer height: 0.16 mm
12	Cable Guide	FDM/PLA	Wall thickness: 1.2 mm Top/bottom thickness: 1.2 mm Infill density: 20 % Layer height: 0.16 mm
13	Traveler Bit	FDM/PLA	Wall thickness: 1.2 mm Top/bottom thickness: 1.2 mm Infill density: 20 % Layer height: 0.1 mm
14	Traveler Bit Holder	FDM/PLA	Wall thickness: 1.2 mm Top/bottom thickness: 1.2 mm Infill density: 20 % Layer height: 0.1 mm
15	Traveler Guide	FDM/PLA	Wall thickness: 1.2 mm Top/bottom thickness: 1.2 mm Infill density: 20 % Layer height: 0.1 mm
16	Traveler Bit Tensioner	FDM/PLA	Wall thickness: 1.2 mm Top/bottom thickness: 1.2 mm Infill density: 20 % Layer height: 0.1 mm
17	Curved Frame Support	FDM/PLA	Wall thickness: 1.2 mm Top/bottom thickness: 1.2 mm Infill density: 20 % Layer height: 0.16 mm
18	Spool Magnet Mount	FDM/PLA	Wall thickness: 1.2 mm Top/bottom thickness: 1.2 mm Infill density: 20 % Layer height: 0.16 mm
19	Electronics Tray	FDM/PLA	Wall thickness: 1.2 mm Top/bottom thickness: 1.2 mm Infill density: 20 % Layer height: 0.16 mm
20	Frame Backing Plate	FDM/PLA	Wall thickness: 1.2 mm Top/bottom thickness: 1.2 mm Infill density: 20 % Layer height: 0.16 mm

Post processing of all parts was conducted by removing support and adhesion material from components and smoothing of printing artifacts using 220 grit sandpaper and/or a fine file.

### 4.2 Insert Installation

**Fig. 1:**
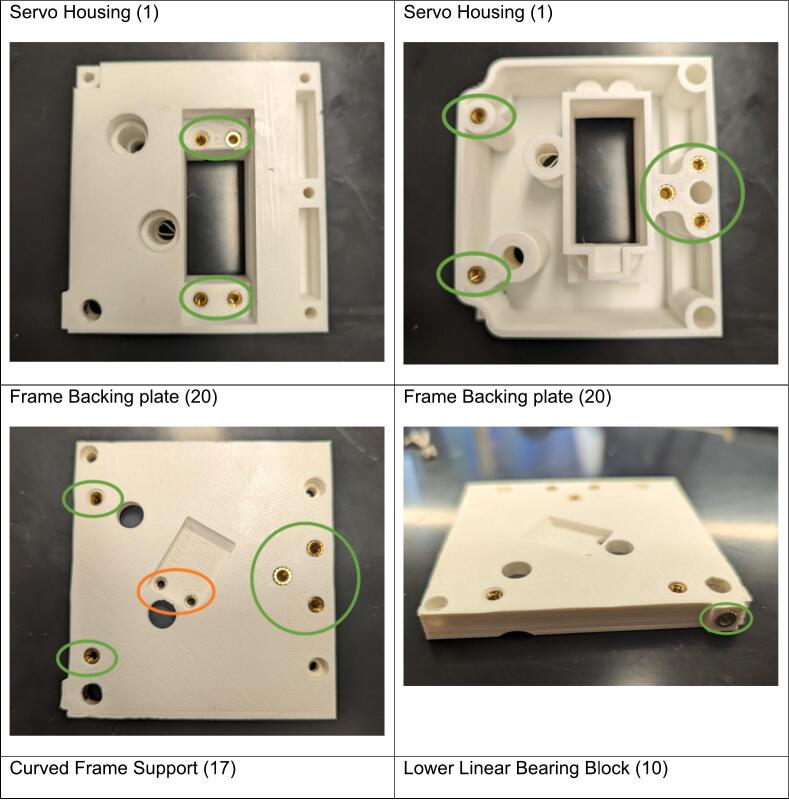

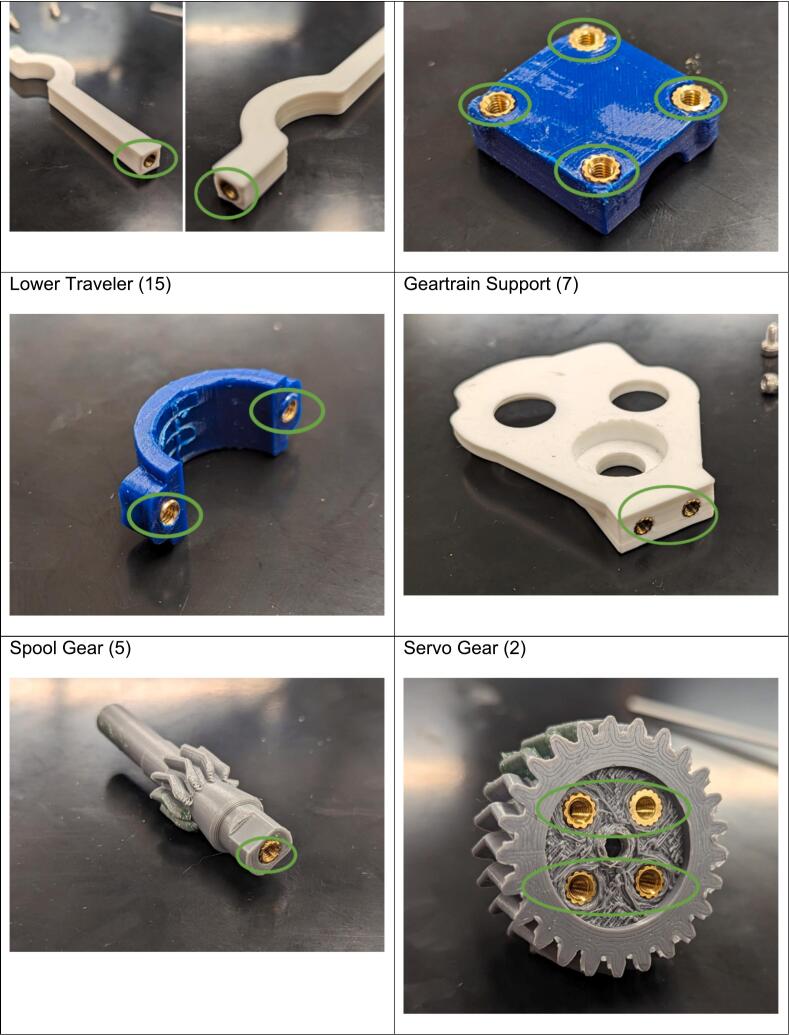
Installation locations for brass inserts in the 3D printed components. Green circles signify M3 brass inserts (M) and orange circles indicate that M2.5 brass inserts (L) should be used. (For interpretation of the references to color in this figure legend, the reader is referred to the web version of this article.)

Add brass inserts to each PLA piece as follows, where green circles indicate M3 (M) brass inserts and orange circles indicate M2.5 (L) brass inserts (Fig. 1):

### 4.3 Mechanical assembly

**Fig. 2:**
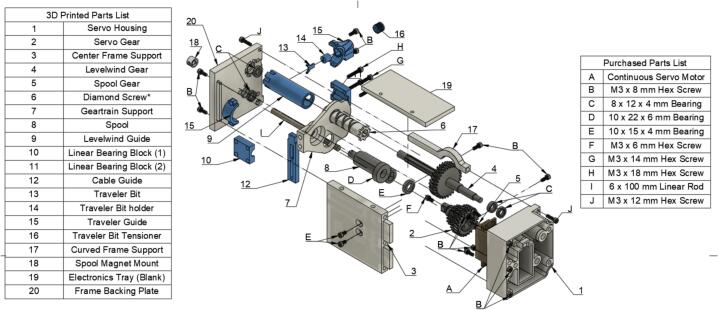
Exploded view of the complete winch hardware assembly. Numbered items (1–20) are 3D printed parts (FDM and *SLA). White parts denote structural housing components (except for the diamond screw), blue represent traveler/guide components for the level wind, dark grey are drivetrain components. Lettered items (A-J) are purchased hardware components (servo, screws, bearings, and linear guide rod). (For interpretation of the references to color in this figure legend, the reader is referred to the web version of this article.)

In the following instructions, the components have generally been color coded by the component they belong to, where white components are parts of the frame, grey parts are part of the geartrain, and blue components are part of the traveler. The exception to this being that the levelwind diamond screw is white SLA printed. A full exploded diagram of the mechanical winch assembly can be found in Fig. 2, and step-by-step instructions follow.

**Fig. 3:**
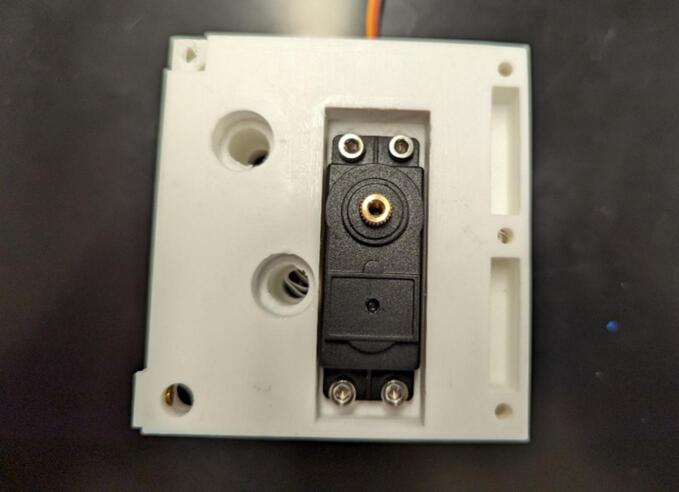
Installation of the continuous servo motor (A) into the motor housing (1).

Begin assembly by removing the rear four screws from the continuous servo motor (A) to allow the motor to pass through the servo housing (1) without damaging the cable and/or stress relief. Pass the motor assembly without the rear cover through the frame servo housing and reattach the rear cover, constraining the servo to be in the housing. Use four M3 x 8 mm screws (B) to attach the servo motor to the housing as shown in Fig. 3.

**Fig. 4:**
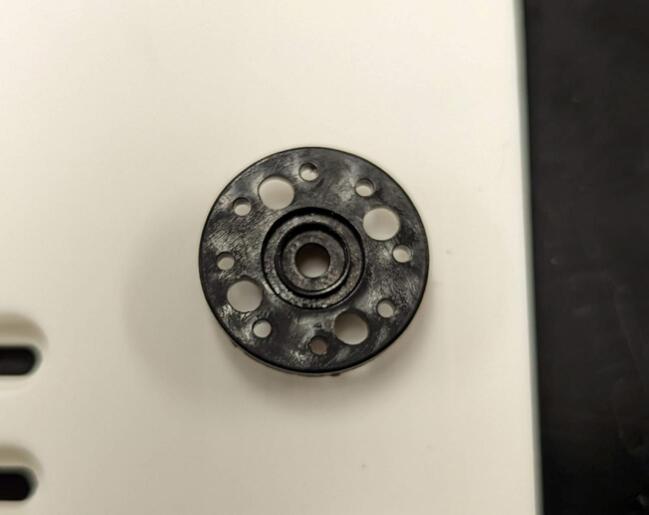
Example of four mounting holes to attach the circular horn (from A) to the servo gear (2).

**Fig. 5:**
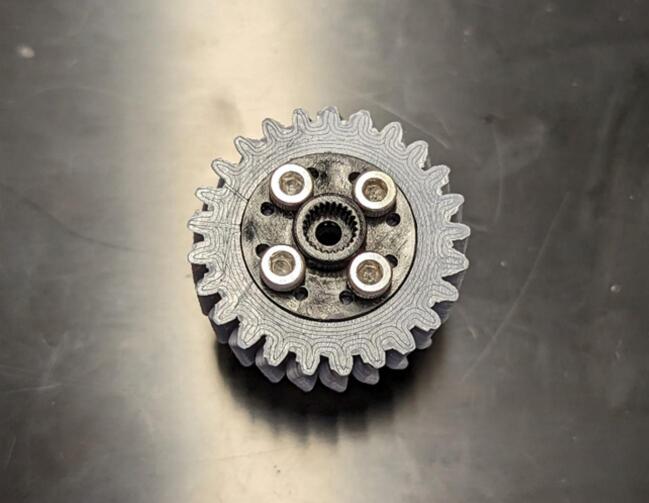
Circular servo horn attached to the servo gear (2).

Drill out the center mounting holes of the circular servo horn (from A) to 3.2 mm (1/8 in) to allow M3 screws to attach the horn to the servo gear (2). Mount the servo horn to the servo gear (2) using four M3 x 6 mm screws (F) (Fig. 4 and Fig. 5).

**Fig. 6:**
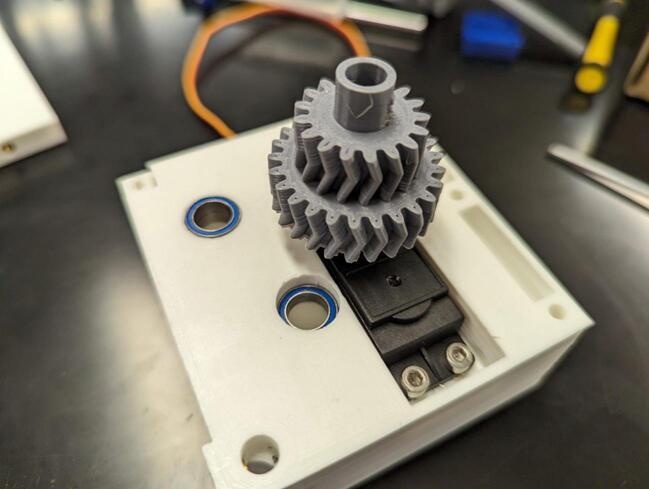
Attachment of servo gear (2) and attached horn to the continuous servo (A). Installation of bearings (C) into the servo housing (1).

Place the servo gear/horn assembly on the servo and attach them loosely using one M3 x 8 mm screw (B). Drop four MR128-2RS bearings (8 x 12 mm) (C) into the spool and levelwind shaft holes on the servo housing (1) (two in each hole). This is depicted in Fig. 6.

**Fig. 7:**
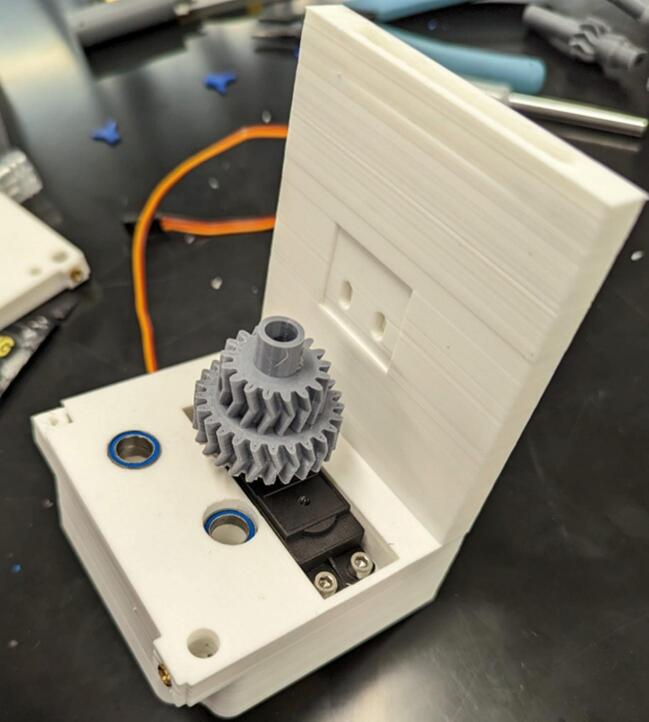
Attachment of the center frame support (3) to the servo housing (1).

Attach the center frame support (3) to the servo housing (1) using three M3 x 8 mm screws (B) as shown in Fig. 7.

**Fig. 8:**
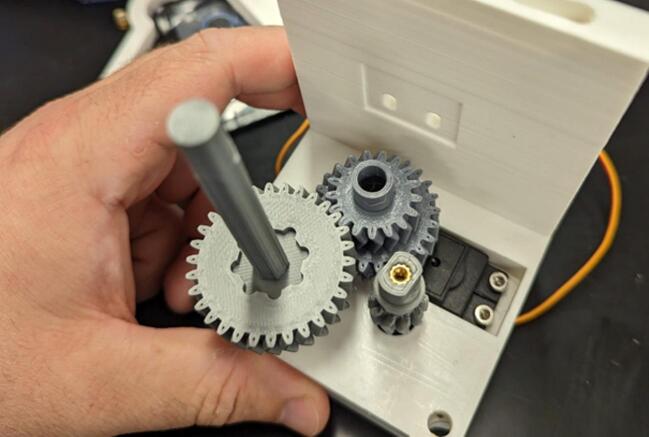
Installation of the levelwind gear (4) and the spool gear (5).

Drop the spool gear (5) into its mounting hole in the servo housing (1), then drop the levelwind gear (4) into its mounting hole in the servo housing as shown in Fig. 8. Tighten down the screw holding the servo gear (2) to the servo (A).

**Fig. 9:**
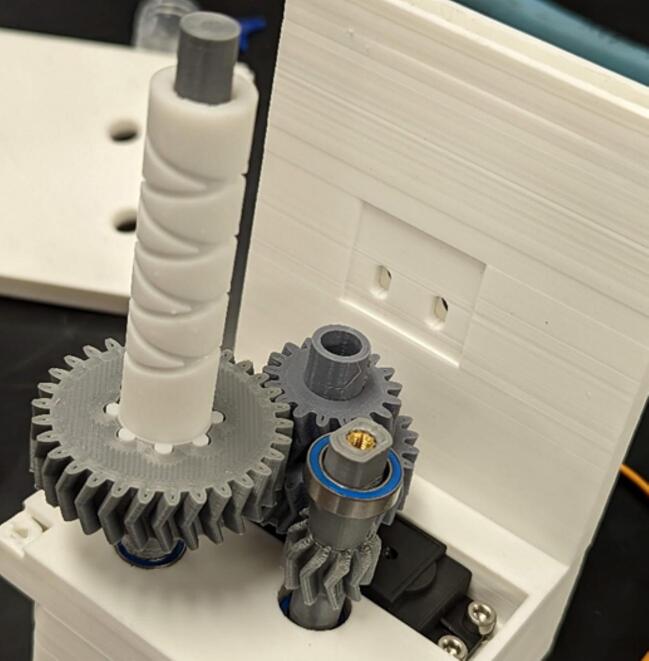
Placement of spool gear (5) bearing and diamond screw (6).

Place one 6700-2RS bearing (10 x 15 mm) (E) on the spool gear (5). Place diamond screw (6) onto the levelwind gear (4), making sure the teeth at the bottom of the diamond screw interlocks with the face of the gear as depicted in Fig. 9.

**Fig. 10:**
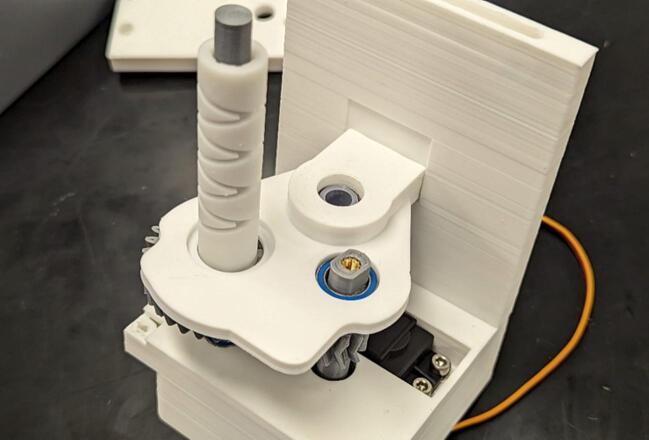
Installation of the geartrain support (7) to the center frame support (3).

Place 10 x 22 mm bearing (D) into geartrain support (7), then add the geartrain support (7) to the assembly, ensuring the bearing faces the servo gear (2). Use two M3 x 6 mm screws (F) to attach the support to the center frame support (3). This is shown in Fig. 10.

**Fig. 11:**
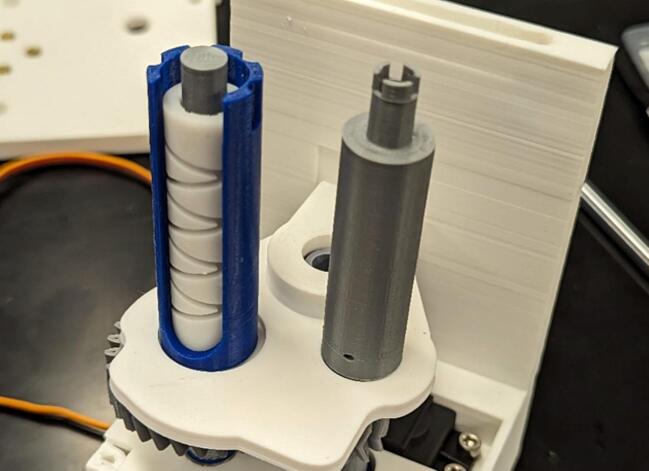
Levelwind guide (9) slides over the diamond screw (6) and the spool (8) is secured to the spool gear (5).

Add spool (8) to spool gear (5) and secure to spool gear with one M3 x 6 mm screw. Add Levelwind guide (9) to diamond screw (6) shown in Fig. 11.

**Fig. 12:**
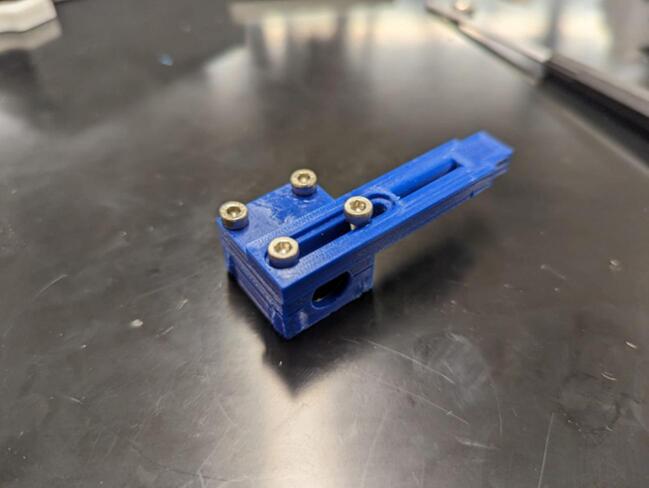
The linear bearing is enclosed in the bearing holder composed of 2 linear bearing blocks (10, 11). The cable guide (12) is then attached as shown.

Place the linear bearing (O) on the linear bearing holder halves (10, 11) and, attaching the cable guide (12) as shown, screw together the linear bearing holder using two M3 x 14 mm screws (G) and two M3 x 18 mm screws (H). This is depicted in Fig. 12.

**Fig. 13:**
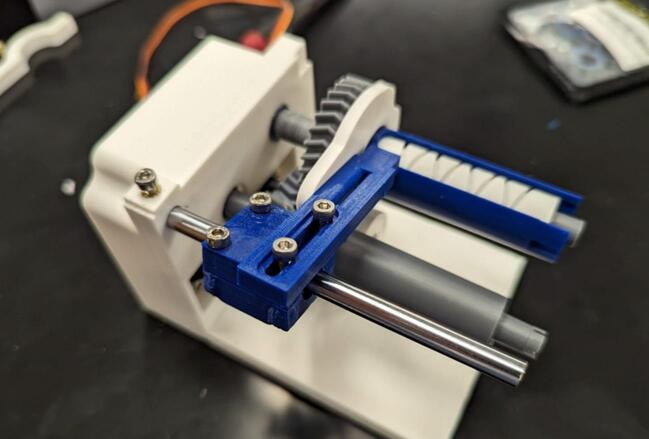
The linear bearing assembly slides onto the linear rod (I) which is affixed to the servo housing (1).

Place linear bearing assembly onto the linear rod (I) and push the linear rod into the servo housing linear rod mount. Secure with one M3 x 8 mm screw (B) (Fig. 13).

**Fig. 14:**
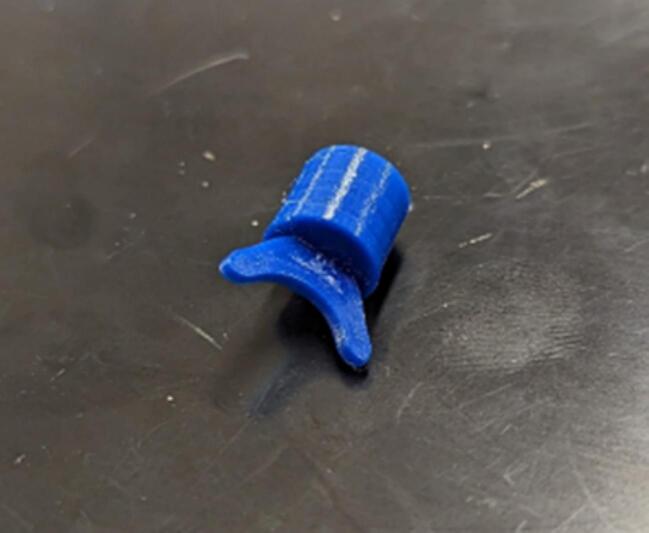
Traveler bit assembly (13 and 14).

**Fig. 15:**
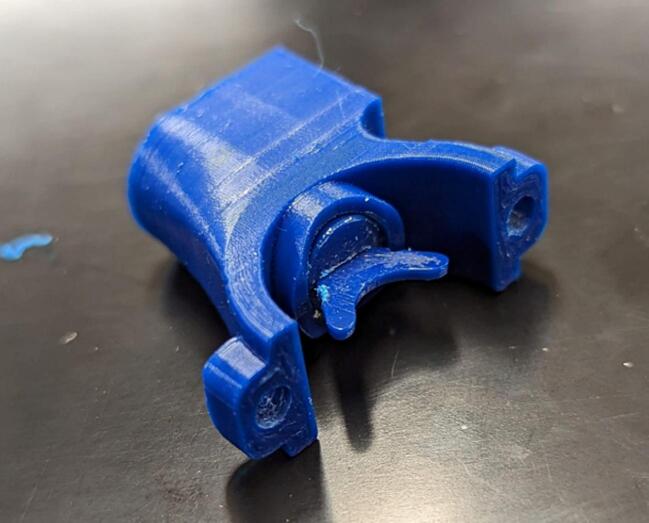
Traveler bit assembly (13, 14) installed in the traveler guide (15).

Push traveler bit (13) into the bit holder (14). Place a small amount of plastic safe general-purpose lubricant (in this case, Magnalube G was used) to the bit assembly, and push the bit assembly into the traveler guide (15) (Fig. 14 and Fig. 15).

**Fig. 16:**
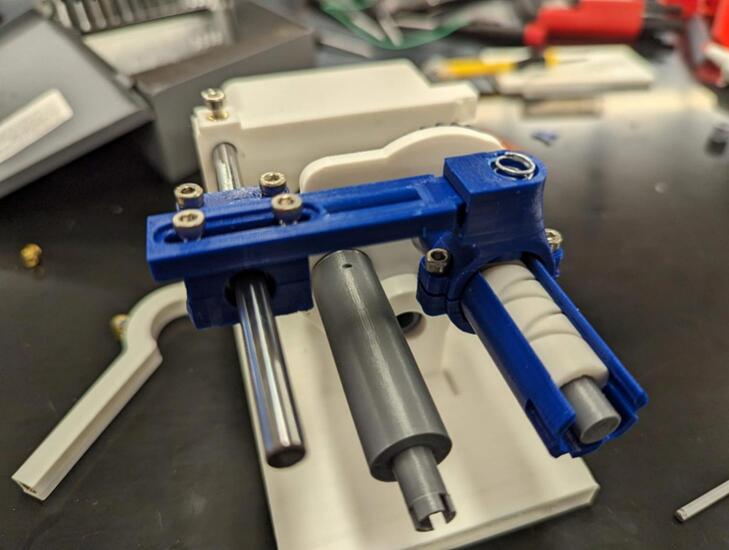
Two halves of the traveler guide (15) slid around the levelwind guide (9) and attached to the cable guide (12).

Attach the traveler guide (15) to the lower traveler using two M3 x 8 mm screws (B) and place the end of the cable guide (12) into the traveler guide. Add the compression spring (P) to the traveler guide (15) as shown in Fig. 16.

**Fig. 17:**
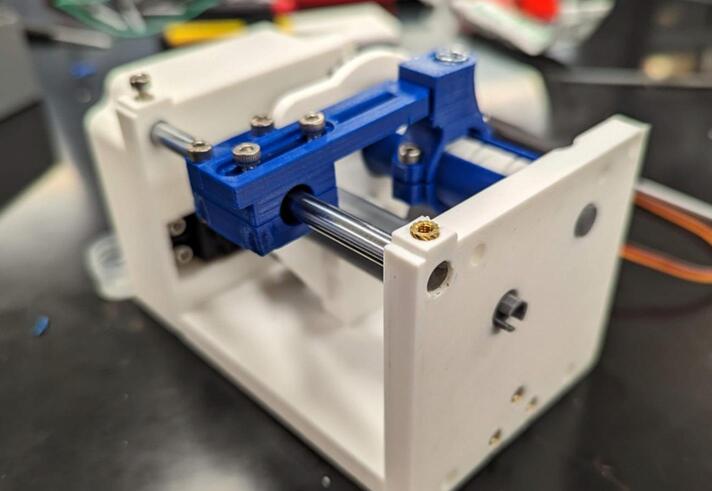
Installation of the back half of the servo housing assembly (1) with bearings (C).

Add two MR128-2RS bearings (8 x 12 mm) (C) to the frame backing plate 20 and attach the plate to the center frame support (3). Fasten the center frame support (3) with two M3 x 14 mm screws (G). Secure the linear rod (I) to the frame backing plate with one M3 x 8 mm screw (B) (Fig. 17).

**Fig. 18:**
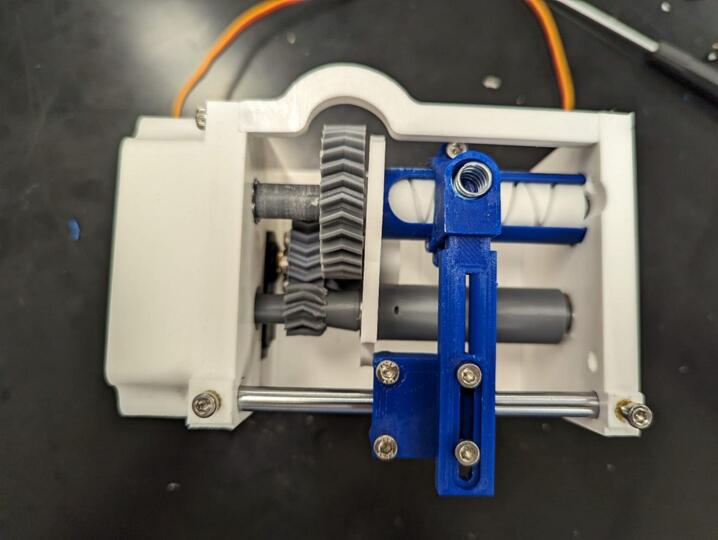
Addition of curved frame support (17).

Add the curved frame support (17) using two M3 x 12 mm screws (J) as shown in Fig. 18.

**Fig. 19:**
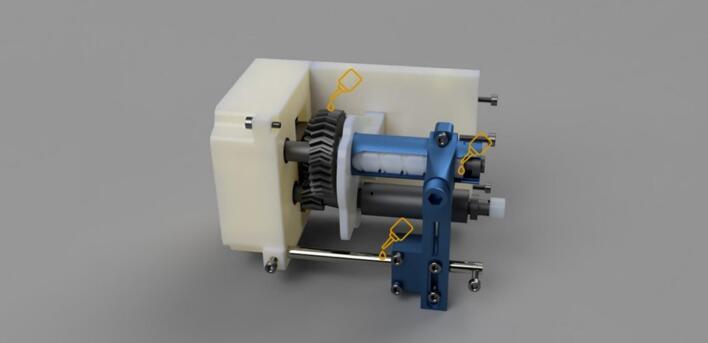
Assembly lubrication guide, with frame backing plate 20, curved frame support (17), and electronics tray (19) removed for visibility.

Place plastic safe lubricant on the gears (2,4,5), linear rod (I), and diamond screw (6) as shown in Fig. 19. Add tensioner screw (16) to traveler assembly. Push the traveler away from the linear rod, adjust the cable guide, tighten the traveler bit tensioner, and tighten the screws securing the guide to the linear bearing assembly.

**Fig. 20:**
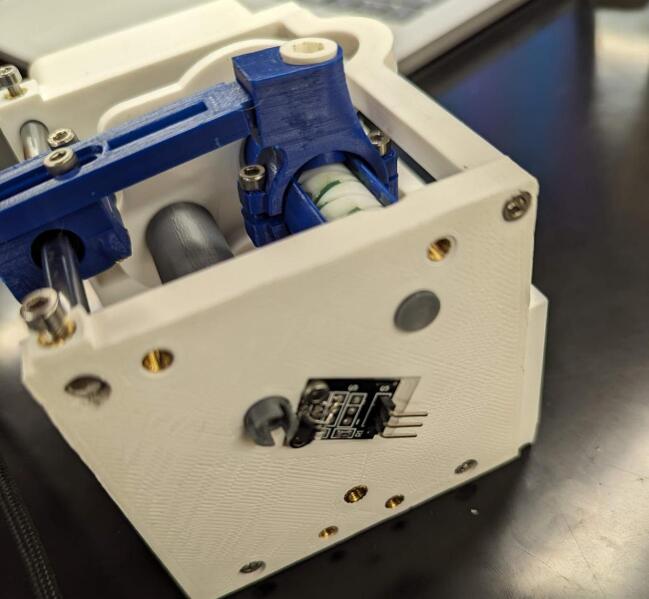
Traveler bit tensioner (16) constraining the traveler spring.

The spring tensioner for the levelwind traveler can now be adjusted using the white hex screw on top of the traveler guide as seen in Fig. 20.

### 4.4 Electronics assembly

The electronics assembly for the CLARK-3 will vary based on the desired payload type and instrumentation. The basic platform supports a hall effect sensor to measure the dispensed line and a limit switch to shut down the motor when the payload has reached the bottom of the winch platform. In the test case in this manuscript, a water sampling container was used as a payload and the winch was automatically shut off when the container contacted the limit switch on the payload collector.

**Fig. 21:**
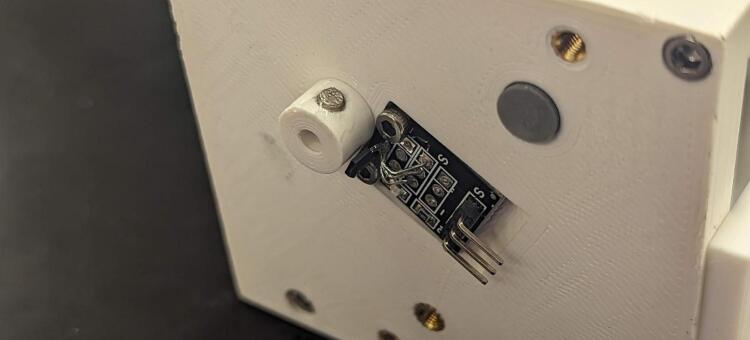
Installation of the hall effect sensor and magnet housing (18) to the back plate of the servo housing (1).

The Hall effect sensor (U3) is attached to the build platform on the back plate using two M2.5 x 8 mm screws (K). The magnet (N) for the Hall effect sensor is mounted to the keyed end of the spool that extends past the backing plate. To ensure accurate counting, power the Hall effect sensor by supplying it 5 V and check which side of the magnet is the south end (the red light on the hall effect sensor will light when the south side is facing the sensor). Using a two-part epoxy, adhere the magnet to the 3D printed spool mount with the south side of the magnet facing outward (Fig. 21).

The limit switch (SW1) can be flexibly mounted based on the payload the user intends to lift. It is mounted with two M2.5 screws (K).

**Fig. 22:**
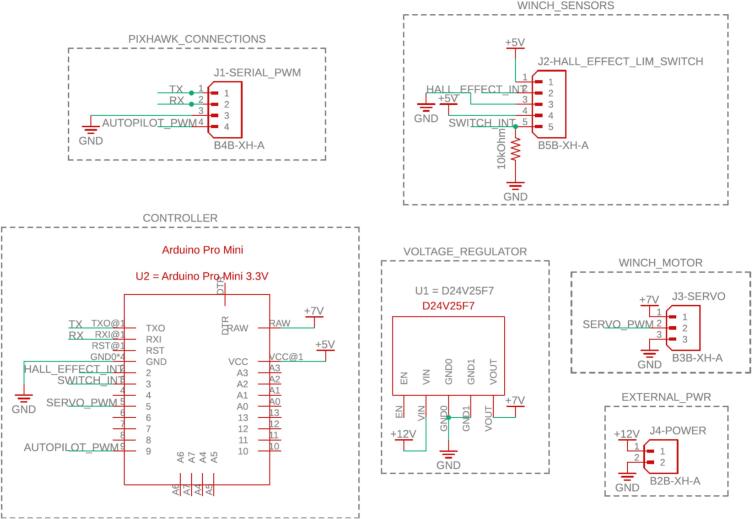
CLARK-3 control board schematic.

**Fig. 23:**
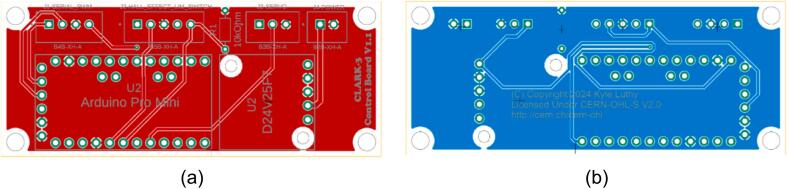
Control board layout (a) top and (b) bottom.

**Fig. 24:**
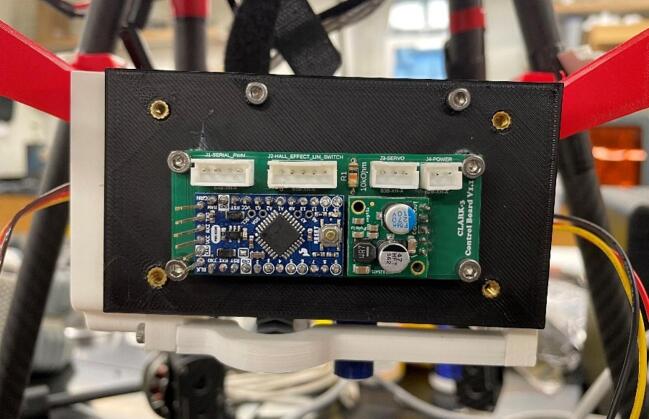
Circuit assembly for winch control mounted to the backing plate.

The control and interface electronics printed circuit board (PCB) can be mounted to the blank electronics tray (19). The blank tray allows for configurable board mounting using M2.5 inserts (L) and M2.5 mm screws (K). This is shown in Fig. 24. Fig. 22 shows the circuit diagram for the CLARK-3 and Fig. 23 depicts the board layout. The board consists of an Arduino Pro Mini 3.3 V (U2), a Pololu voltage regulator (U1), and headers (J1-J4) to support connection to the autopilot/controller, the limit switch, hall effect sensor, and the winch servo. The Pololu regulator is a 7.5 V, 2.4A step-down regulator which is supplied directly from the drone’s battery (up to 38 V). The corresponding cables mating to J1 through J4 are not depicted as these will be custom to the system that the CLARK-3 is being integrated with. Lengths will be dependent on the airframe geometry and sensor mounting locations. Receptacles for the CLARK-3 board interface cables have been specified (J1-A through J4-A) but the receptacles at the other end of the cables will depend on the headers native to the autopilot or other controller of the system.

### 4.5 Software

**Fig. 25:**
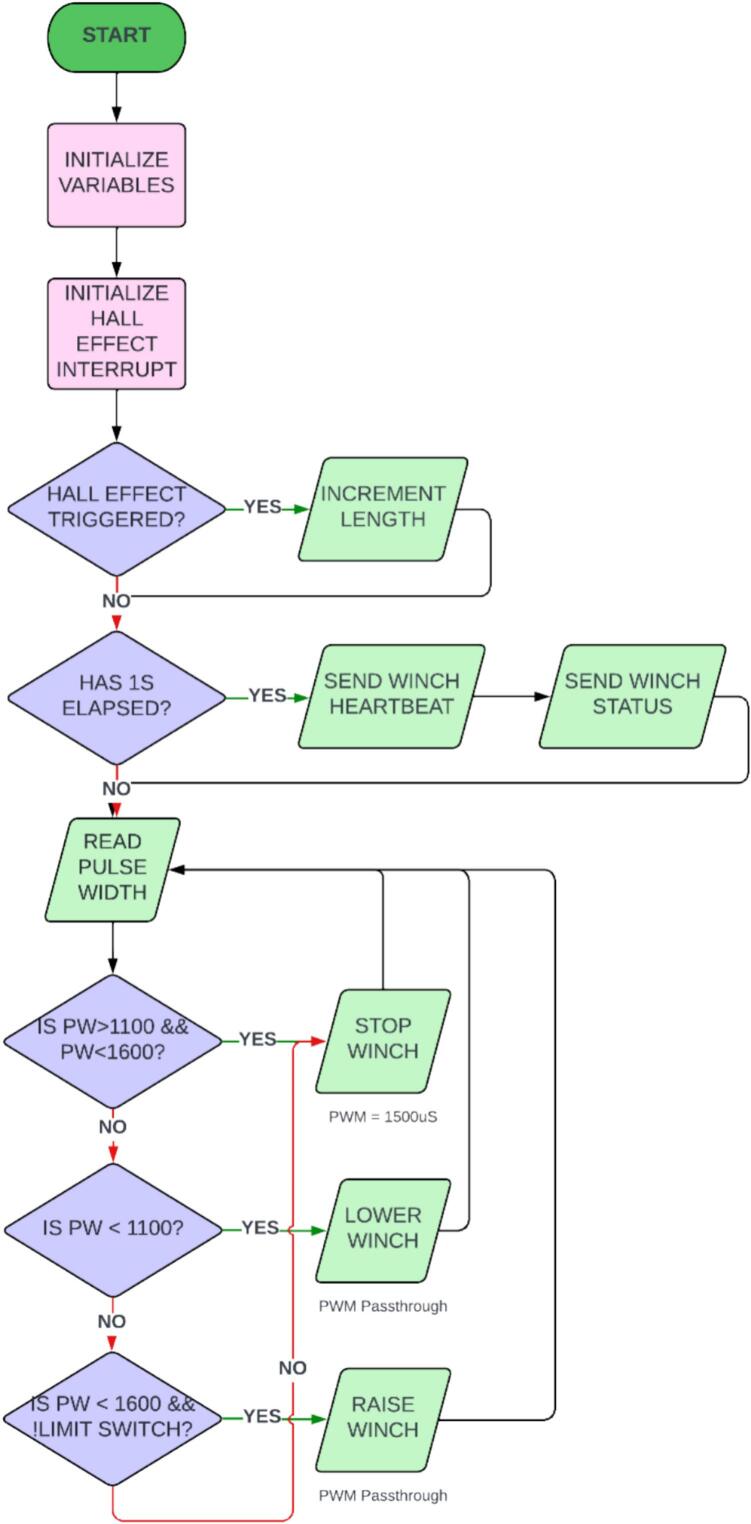
Flowchart of Arduino code to control the state of the winch with an integrated limit switch indicator and Hall Effect count for line deployment feedback.

This system is customizable, and the software configuration will vary depending on the autopilot, the physical controller, and any ground control software. Ground control software is only needed if line payout feedback from the integrated Hall effect sensor is desired. If this metric is not required, it can be ignored, and the sensor and magnet do not need to be installed on the system. However, this is not recommended as adjustable safety limits can be defined in the code to account for over retraction of line or line deployment past the end of its length. It is possible to have this information relayed directly to the flight remote, but this will be highly dependent on the controller that is in use and may require additional hardware modifications to the control radio. The system test setup uses a MavLink [27] library on the Arduino Pro Mini (U2) to relay the Hall effect sensor (U3) count via telemetry through a PixHawk 2 autopilot to a computer running ground control software such as QGroundControl [28] or Mission Planner [29]. Regardless of the hardware and software used, the winch can be integrated if it has access to a controllable PWM output on the platform. For this application, a three-way switch on the remote control (a Taranis Q X7) was used to select the state of the winch motor: stopped, up, and down. The winch speed corresponding to the winch direction is programmed into the autopilot directly and this procedure may vary between systems. By default, the code running on the interface board (PCB) will pass through the PWM from the autopilot directly to the winch servo. However, this could be altered to adjust the speed as well. Additional switches could be programmed to alter the PWM duty cycle and provide additional speed control options. The digital limit switch also forces the system into the stopped state when it is triggered while the motor is in the up state. A flow chart of the Arduino code is shown in Fig. 25.

## 5 Operation instructions

Mounting the winching platform to the drone will vary by drone, but generally the winching platform will require custom-built mounting hardware like the hardware provided in the files labeled Sample_Drone_Mounting_Hardware. The winching platform has three threaded holes provided on both the servo housing and backplate, to ease mounting. Connections to the drone, as described above, are simply connecting to an 8.3–38 V DC power supply to the Pololu voltage regulator and connecting the PWM signal from a Pixhawk (or similar) auxiliary channel to the Arduino input.

To load line onto the winching platform spool, begin by feeding line through the retaining hole in the spool and actuate the winch to wrap the line around the spool, maintaining light tension on the line as it is loaded. Ensure the loose end of the line is appropriately attached to the sampling container to engage the limit switch as the end of the line is pulled up by the winch; this ensures that the winch line is kept in some tension when it is in flight and prevents the payload from acting as a pendulum independent of the drone chassis. Due to the scale and strength of this winching platform, the hazards are relatively limited, but there is a trapping hazard due to the moving geartrain of the winch, and long hair or loose clothing articles should be kept away from the winch.

Once the multirotor and transmitter have been configured by the user, the winching platform operates fairly simply. A channel configured to control the raise/lower action of the CLARK-3 may be used by the operator without concern for damaging the drone due to the limit switch of the winching platform. In addition, loads excessively heavy for the winch geartrain will slowly lower when the winch is in the neutral state; in this case, the operator can leave the winch in the “raise” position, causing the CLARK-3 to briefly raise the payload each time the payload drops below the limit switch. This function keeps the payload engaged with the chassis and improves the handling of the drone. As a safety measure, the end of the winch line is not well-secured to the winch spool; if the payload gets trapped while it is lowered and the drone is not able to free the payload, the operator can pay out all of the line under throttle and release the drone from the trapped payload.

## 6 Validation and characterization

**Fig. 26:**
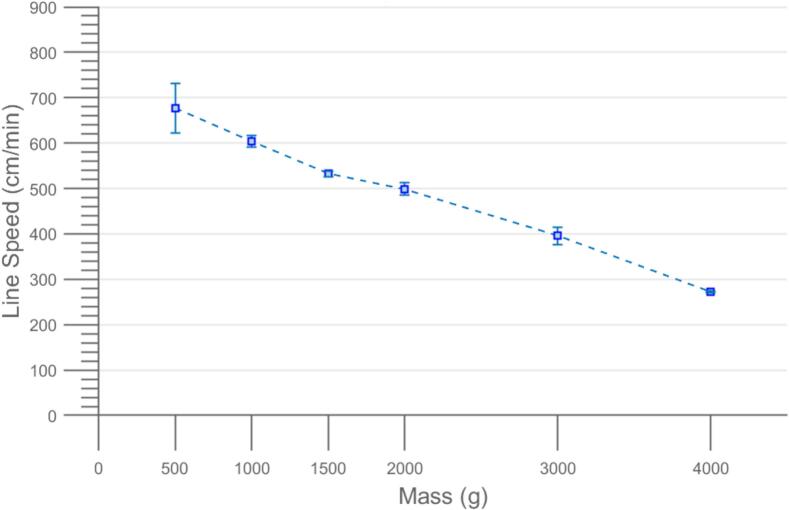
The results of benchtop testing of the CLARK-3 winching platform. 500 g, 1000 g, 1500 g, 2000 g, 3000 g, and 4000 g of payload were tested resulting in 676.1 ± 54.8 cm/min, 604.4 ± 13.0 cm/min, 532.2 ± 5.82 cm/min, 498.6 ± 13.9 cm/min, 395.6 ± 19.1 cm/min, 271 cm/min.

Validation of the CLARK-3 was conducted first via bench testing on a custom fabricated testing platform. The Arduino controlling the winching platform was triggered via a function generator at the maximum speed (2 ms pulse) to validate function and weights were incrementally added to a 130 N monofilament. Testing was conducted on 0.5, 1, 1.5, 2, 3, and 4 kg payloads by lifting then lowering them 100 m. Line speed was evaluated by raising and lowering payloads three times with a scale background and conducting video analysis to measure average line speed during lifting and means and standard deviations are reported as shown in Fig. 26. In testing, little system strain was noted at payloads of 2 kg and lower, with the system maintaining about 500 cm/min of line speed or greater. With a payload of 3 kg the speed at which the system lifted was slightly reduced to 395 cm/min, but 100 m of line was successfully raised and lowered three times without system failure. Using a 4 kg payload resulted in further reduced lifting speed to 271.9 cm/min, and failure occurred during the second raising of the payload 100 m (at approximately 120 m). It should be noted that “failure” in this context did not result in the winching system breaking and falling from the drone, but rather the 3D printed connection between the spool gear and the spool breaking loose and allowing the payload to freewheel to the end of the line. This could be improved by increasing the clamping load between the spool gear and spool or by revising the material used for these components.

**Fig. 27:**
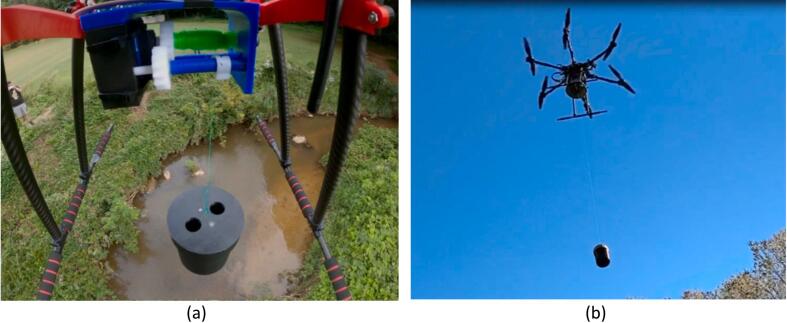
Field testing of the CLARK-3 winch with (a) view of water sampler attached to the winch over a stream surrounded by heavy vegetation and (b) water sampling payload being deployed with the winch.

After bench testing was completed, Field testing was conducted using a custom built hexacopter with a total payload capacity of 10 kg. For field testing, the PWM signal was programmed to an auxiliary controller switch using QGroundControl and Pixhawk signal output. Custom mounting hardware was 3D printed to attach the winching platform to the landing gear of the multirotor and a custom sampling container was added specifically for remote water sampling tasks. Field testing was conducted at Lake Katharine, part of the Reynolda House in Winston-Salem, NC. The test included water sampling in a stream near a wooded area, representing a potentially difficult to sample area. Images from the water sampling trial are shown in Fig. 27.

## 7 Conclusion

The CLARK-3 winching platform has been successfully bench and field tested. In bench testing, the system was able to raise and lower payloads of up to 4 kg without failure. In field testing, this system was used to successfully collect water from a stream while minimizing prop wash on the stream surface.

Future work on the CLARK-3 could focus on improving the maximum payload or speed of the winch or adding application-specific features.

Key Takeaways.

• Modular design – allows for easy customization or integration with various drone platforms or payloads
• Benchtop testing – demonstrated reliable lifting at payloads even higher than rated
• Field Testing – demonstrated lowering and lifting of a custom water sampling vessel into a stream
• Safety Features – integrated limit switch prevents over-retraction and helps to ensure safe operation
• Straightforward Assembly – Using approachable 3D printing techniques and limiting advanced materials, most labs should be able to assemble and use this winching platform

## CRediT authorship contribution statement

**William Crowe:** Writing – review & editing, Writing – original draft, Visualization, Validation, Software, Methodology, Formal analysis, Conceptualization. **Benicio Costales:** Writing – review & editing, Software, Methodology, Conceptualization. **Kyle Luthy:** Writing – review & editing, Writing – original draft, Visualization, Validation, Supervision, Software, Methodology, Conceptualization.

## Funding

This work was supported by internal funding from Wake Forest University.

## Declaration of competing interest

The authors declare that they have no known competing financial interests or personal relationships that could have appeared to influence the work reported in this paper.
